# The Role of CXCR2, MMP-2, and MMP-9 in the Pathogenesis of Placenta Accreta: A Molecular Expression Study

**DOI:** 10.3390/medicina61030461

**Published:** 2025-03-06

**Authors:** Putri Mirani, Krisna Murti, Peby Maulina Lestari, Iche Andriyani Liberty, Cindy Kesty, Hana Andrina, Bella Stevanny

**Affiliations:** 1 Division of Maternal-Fetal Medicine, Department of Obstetrics and Gynecology, Dr. Mohammad Hoesin General Hospital, Faculty of Medicine, Universitas Sriwijaya, Palembang 30114, South Sumatra, Indonesia; putrimirani@fk.unsri.ac.id (P.M.); pebymaulinalestari@fk.unsri.ac.id (P.M.L.); 2 Department of Anatomic Pathology, Dr. Mohammad Hoesin General Hospital, Faculty of Medicine, Universitas Sriwijaya, Palembang 30114, South Sumatra, Indonesia; 3 Department of Public Health and Community Medicine, Faculty of Medicine, Universitas Sriwijaya, Palembang 30114, South Sumatra, Indonesia; iche.aliberty@gmail.com; 4 Department of Obstetrics and Gynecology, Dr. Mohammad Hoesin General Hospital, Faculty of Medicine, Universitas Sriwijaya, Palembang 30114, South Sumatra, Indonesiahana.andrina@gmail.com (H.A.); bellastevanny@student.unsri.ac.id (B.S.); 5 World Health Organization, Tropical Diseases Research, Clinical Research Leadership Fellow, Infectious Diseases Data Observatory, University of Oxford, Oxford OX3 7LF, UK; 6 National Task Force of Reproductive Tract Infection, Indonesian Society of Obstetrics and Gynecology, Jakarta 10320, Indonesia

**Keywords:** CXCR2, immunohistochemistry, MMP-2, MMP-9, Placenta accreta spectrum disorder

## Abstract

*Background and Objectives*: The pathogenesis of placenta accreta spectrum disorder (PASD) is influenced by the inflammatory process. Therefore, the examination of biomarkers related to the inflammatory process, namely matrix metalloproteinase (MMP) and CXC motif chemokine receptor 2 (CXCR2), is expected to bring researchers to a bright spot regarding the pathogenesis of PASD. This study analyzes the role of CXCR2, MMP-2, and MMP-9 in the pathogenesis of PASD. *Materials and Methods*: An observational study with a case–control design was conducted to assess differences in the mean density of CXCR2, MMP-2, and MMP-9 immunostaining in placental and uterine tissue in 17 patients with PASD and 34 patients without PASD at the Department of Obstetrics and Gynecology, Dr. Mohammad Hoesin Hospital Palembang. The expression of CXCR2, MMP-2, and MMP-9 was measured by immunohistochemistry analysis. The data were analyzed using STATA version 15. *Results*: There were no significant differences in the mean levels of MMP-2 expression in patients with and without PASD. There were significant differences in the expression of placental CXCR2 (*p* = 0.003), uterine CXCR2 (*p* < 0.001), and uterine MMP-9 (*p* = 0.018) in patients with and without PASD. *Conclusions*: CXCR2 and MMP-9 may play a role in the pathogenesis of PASD.

## 1. Introduction

Placenta accreta spectrum disorder (PASD), an abnormal attachment of the placenta to the uterine wall, is an increasingly prevalent complication of pregnancy associated with significant maternal morbidity and mortality [1]. Globally, PASD occurs in approximately 1:1000 deliveries, having increased from 0.04% to 0.9% over the last 20 years [2]. There is no overall incidence of PASD in Indonesia, but there has been an increase in incidence at Dr. Soetomo Regional General Hospital Surabaya from 0% in 2014 to 2% in 2016, which is predicted to continue to increase [3]. Research by Desmalia et al. [4] reported that the prevalence of accreta among all pregnant women in 2018–2021 was 0.08% (2 out of 2424 pregnant women); 0.21% (4 out of 1910 pregnant women); 1.72% (23 out of 1336 pregnant women); and 2.8% (36 out of 1285 pregnant women) at Dr. Mohammad Hoesin General Hospital Palembang, respectively [4]. In a study at Dr. Zainoel Abidin Hospital Banda Aceh, the incidence of PASD in 2019–2020 was 2.3% [5]. This disorder is a contributor to maternal mortality due to the postpartum hemorrhage that occurs. PASD increases maternal morbidity up to 18-fold and is most commonly associated with massive postpartum hemorrhage. The mortality rate from PASD is approximately 7% but increases to 30% if not diagnosed antenatally due to attempts to separate the placenta after delivery [6].

While the mechanical factors, such as previous cesarean delivery and surgical history, have been extensively studied, the molecular mechanisms underlying PASD remain underexplored. Recent advances in the understanding of placental biology suggest that inflammatory processes may play a critical role in the development of this condition. Particularly, the expression of biomarkers such as CXC motif chemokine receptor 2 (CXCR2) and matrix metalloproteinases (MMP-2 and MMP-9) in the uterus and placenta appears crucial yet insufficiently studied. Cell movement across the extracellular matrix (ECM) and the modification and breakdown of the ECM by MMPs are crucial components of the wound healing process. Matrix metalloproteinases are a collection of enzymes that include zinc (Zn) and require calcium to function. They play a role in breaking down the extracellular matrix (ECM). MMP-2 has a role in wound healing by accelerating cell migration, while MMP-9 is secreted by keratinocytes at the leading edge of the wound to promote cell migration and the development of new epithelial tissue. Both MMP-2 and MMP-9 are present in the damaged epithelium [7]. Matrix metalloproteinases play a vital role as enzymes in the initial stages of pregnancy by facilitating trophoblast cell penetration and invasion. This is supported by the research of Chen et al. [8], who reported a higher positive level of MMP-9 expression in the PASD group compared with the control group. Research by El-Hussieny et al. [9] also reported that MMP-2 staining in the cytoplasm of trophoblast villus and extravillous placental tissue of PASD patients was higher than normal placenta. The biomarker CXCR2 is involved in trophoblast invasion, neovascularization, and vascular remodeling processes [10]. Women with pre-eclampsia exhibit less CXCR2 expression in their placental tissue compared to those with normal pregnancies [11]. In PASD, the levels of immunostaining expression are likely to be elevated compared to a standard pregnancy due to the overexpression of CXCR2. The study by Ma et al. [12] also reported that CXCR2 as a CXCL1 receptor promotes endothelial cell proliferation, migration, and angiogenesis and plays a role in decidual angiogenesis during the first trimester of pregnancy.

Despite the recognized importance of these markers in other obstetric pathologies, there is a conspicuous lack of data regarding their roles in PASD. CXCR2, a key mediator in inflammatory and angiogenic responses, has been implicated in various placental functions but its specific contributions to the pathogenesis of PASD have not been adequately addressed in the existing literature. This gap highlights a critical need for focused research to elucidate the potential diagnostic and therapeutic implications of CXCR2, along with MMP-2 and MMP-9, in managing PASD. Our study aims to fill this gap by providing a detailed molecular expression analysis of CXCR2, MMP-2, and MMP-9 in placental and uterine tissues of PASD patients compared to controls. By focusing on these biomarkers, we seek to uncover potential mechanisms that could explain the invasive nature of the placenta in PASD, offering insights that could lead to better clinical outcomes through targeted therapies. This research is not only necessary for a deeper understanding of PASD at the molecular level but also pivotal for developing preventive and management strategies.

## 2. Material and Methods

### 2.1. Study Population

This case–control study involves 51 people, comprising 17 PASD patients and 34 healthy controls. The minimum sample size was determined using the sample size estimate formula for analytical research with numerical data in order to ascertain the mean difference between two independent groups. Using a confidence level of 95% (α = 0.05, zα = 1.96), a power level of 90% (β = 0.1, zβ = 1.28), and a total proportion of 35% from a previous study by Chen et al. [8], a minimum sample size of 15 was obtained. The recruitment of study subjects was carried out using the consecutive sampling method. With a case–control ratio of 1:2, a minimum of 51 subjects was required, consisting of 17 patients with PASD and 34 patients without PASD. The study population consisted of pregnant women who had previously undergone a caesarean section with and without PASD and visited Dr. Mohammad Hoesin Hospital Palembang between June and December 2023. The inclusion criteria for the case group included pregnant women with PASD. The control group consisted of expectant mothers with a history of a caesarean section without PASD and who were not yet in labor. The exclusion criteria included pregnant women with multiple fetus and pre-eclampsia, and those who refused to participate in this study.

### 2.2. Data Collection

Basic subject characteristics were collected from electronic medical records and medical history taking. The blood sample along with placental tissue was obtained during the third trimester of pregnancy. PASD was detected using 2-dimensional ultrasonography using GE^®^ Voluson™ E-6 (General Electric, Vienna, Austria) and Samsung^®^ RS80A (Samsung Medison, Seoul, Republic of Korea).

The expression levels of MMP-2, MMP-9, and CXCR2 were assessed by immunohistochemical analysis. Five small placental tissue pieces of approximately 1 × 1 × 1 cm in size were cut from the newborn placenta at the central zone (avoiding blood vessels and/or calcium deposits), i.e., at the midpoint between the cord insertion point and the edge of the placental disc. Uterine tissues were collected from patients undergoing surgery for PASD at the time of cesarean delivery or hysterectomy. Immediately following excision, tissues were placed in ice-cold saline and transported to the laboratory. Within 30 min of collection, samples were washed with phosphate-buffered saline to remove excess blood, sectioned into 1 cm^3^ pieces, and fixed in 10% formalin for 24 h at room temperature for subsequent histological examination. Fixed tissues were then embedded in paraffin, sectioned at 4 µm thickness, and processed for immunohistochemical analysis as described for placental tissues.

Immunohistochemical staining was performed to assess the expression levels of MMP-2, MMP-9, and CXC2 in placental and uterine tissues. Tissues were first fixed in 10% buffered formalin and embedded in paraffin before sectioning at 4 µm thickness. For antigen retrieval, slides were heated in a citrate buffer (pH 6.0) at 95 °C for 20 min. After cooling, endogenous peroxidase activity was blocked with 3% hydrogen peroxide in methanol for 10 min at room temperature. Non-specific binding was minimized by incubating the slides with 5% bovine serum albumin (BSA) for 30 min. Overnight incubation at 4 °C was performed with primary antibodies against MMP-2 (1:100 dilution, Abcam, Cat No. ab12345), MMP-9 (1:100 dilution, Abcam, Cat No. ab6789), and CXCR2 (1:50 dilution, Santa Cruz Biotechnology, Cat No. sc-12345) followed by biotinylated secondary antibodies for 1 h at room temperature. These antibodies were selected after extensive preliminary testing to determine the optimal dilution that provides the best specificity and intensity for staining in our tissue samples. Detection was carried out using the Vectastain Elite ABC Kit (Vector Laboratories), and the signal was developed with 3,3′-diaminobenzidine (DAB). Counterstaining was performed using Mayer’s hematoxylin. The examination was conducted at the Anatomical Pathology Laboratory of Barokah Palembang and the Department of Anatomical Pathology of Dr. Mohammad Hoesin Hospital Palembang. Each immunostained slide was digitally scanned at 40× magnification using a Nikon Eclipse 80i microscope (Tokyo, Japan). Quantitative analysis of staining intensity and cell positivity percentage was conducted using ImageJ software 1.54f version (NIH, Bethesda, MD, USA) by analyzing five random fields per slide. The immunohistochemical scoring system integrated both the intensity of the staining (graded as 0: no staining—blue, 1: weak—bluish brown, 2: moderate—light brown, 3: strong—dark brown) and the percentage of positive staining cells (0%: 0, 1–10%: 1, 11–50%: 2, 51–80%: 3, 81–100%: 4). Scores from each field were averaged to obtain the final score for each sample. In order to ensure consistency and reduce inter-observer variability, all immunohistochemical analyses were conducted by the same experienced pathologist, who was blinded to the clinical information of the samples. This standardization in interpretation is critical for maintaining objectivity and reliability in assessing staining patterns and intensity across all samples.

Prior to their enrollment in this study, each participating woman provided written informed consent. This study received ethical approval from the Ethics Committee of the Faculty of Medicine, University of Sriwijaya (DP.04.03/D.XVIII.6.11/ETIKRSMH/14/2023).

### 2.3. Statistical Analysis

All data were analyzed using STATA version 15 (StataCorp LLC, College Station, TX, USA). Univariate analysis was on the basic characteristics of the subjects, which included maternal age, parity, gestational age, and comorbidities. Data were presented descriptively with frequency and percent for categorical data (nominal and ordinal) and mean and standard deviation for numerical data (interval and ratio). Bivariate analysis of the association of each variable with the incidence of PASD was performed through bivariate tests using the Pearson chi-square test with Fisher’s exact alternative for categorical data and an independent T-test with a Mann–Whitney alternative for numerical data. Correlation tests with the Pearson correlation test and Spearman rho test were performed to assess the correlation of calcium ion levels with CXCR2, MMP-2, and MMP-9.

## 3. Results

### 3.1. Characteristics of Research Subjects

A case–control diagnostic study was undertaken at the Obstetrics and Gynecology Department of Dr. Mohammad Hoesin General Hospital Palembang. A total of 51 pregnant women met the inclusion criteria as research subjects. There were 17 pregnant women in the case group and 34 pregnant women in the control group.The study subjects had an average age of 31.82 ± 4.68 years, the majority aged 20–35 years (72.5%) with a BMI of 26.83 ± 3.27 kg/m^2^. Most of the study subjects were multiparous pregnant women (66.7%) with fullterm gestational age (52.9%) and did not have a comorbidity, such as diabetes mellitus (DM) or hypertension (96.1%). The gestational age in the case group was significantly younger than the control group (*p* = 0.005). Meanwhile, the general characteristics of the study subjects, such as maternal age, BMI, parity, and comorbidities between the two groups, were not significantly different. The general characteristics of the study subjects in each group are presented in Table 1.

The PASD group had a higher proportion of subjects aged 20–35 years (76.5%), multiparous (76.5%), and preterm (76.5%) than the non-PASD group. The non-PASD group had a higher proportion of subjects with obesity I (70.5%), obesity II (23.5%), at fullterm (67.6%), and with no comorbidities (97.1%) than the PASD group.

### 3.2. Expression of CXCR2, MMP-2, and MMP-9 in Placental and Uterine Tissue

**Figure 1:**
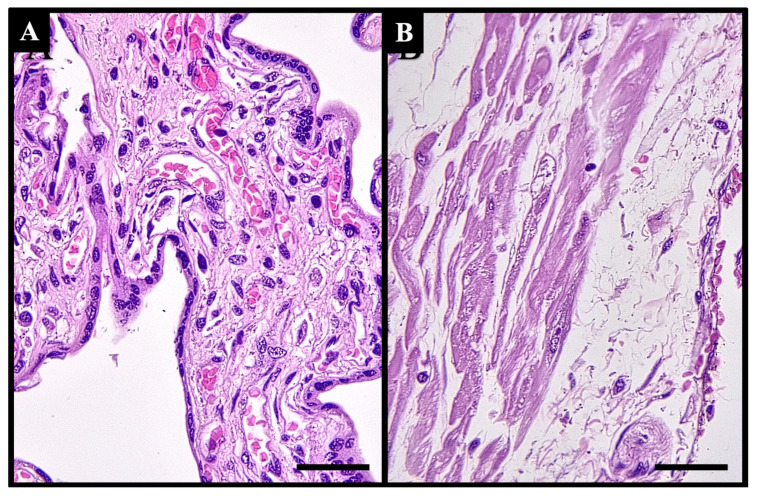
Histological features of (**A**) placenta and (**B**) uterus tissues stained with hematoxylin and eosin before immunohistochemical staining. Scale bar 500 µm; original magnification 400×. This figure was generated using Microsoft PowerPoint, Microsoft, Washington, DC, USA. All images presented were obtained exclusively for this study and have not been previously published or used elsewhere.

**Figure 2:**
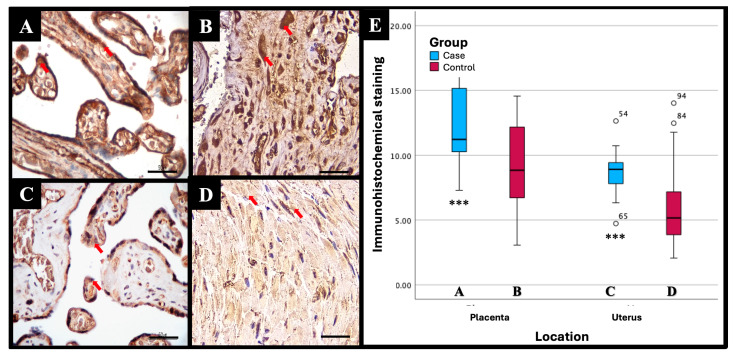
Representative immunohistochemical staining results of CXCR2 with different intensities in (**A**) placenta of PASD group: strong, (**B**) uterus of PASD group: strong, (**C**) placenta of non-PASD group: moderate, and (**D**) uterus of non-PASD group: moderate. CXCR2 expression is characterized by brown staining in the cytoplasm and cell membrane (red arrow); light blue staining indicates hematoxylin-stained nuclei. (**E**) Comparison of mean immunostaining score of CXCR2 in PASD and non-PASD groups. *** Independent *t*-test, *p* < 0.05 compared with the control group. Scale bar 500 µm; original magnification 400×. This figure was generated using Microsoft PowerPoint, Microsoft, Washington, DC, USA. All images presented were obtained exclusively for this study and have not been previously published or used elsewhere.

**Figure 3:**
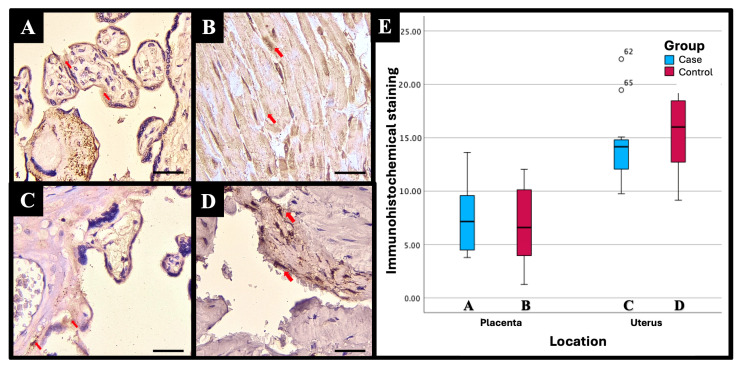
MMP-2 immunohistochemical staining results with different intensities in (**A**) placenta of PASD group: moderate, (**B**) uterus of PASD group: moderate, (**C**) placenta of non-PASD group: weak, and (**D**) uterus of non-PASD group: moderate. MMP-2 expression is characterized by brown staining in the cytoplasm and cell membrane (red arrow); light blue staining indicates hematoxylin-stained nuclei. Note the increased MMP-2 staining in the intervillous spaces compared to control tissues, suggesting enhanced proteolytic activity associated with PASD. (**E**) Comparison of the mean immunostaining expression level (percentage of positive cells) of MMP-2 in PASD and non-PASD groups. Scale bar 500 µm; original magnification 400×. This figure was generated using Microsoft PowerPoint, Microsoft, Washington, DC, USA. All images presented were obtained exclusively for this study and have not been previously published or used elsewhere.

**Figure 4:**
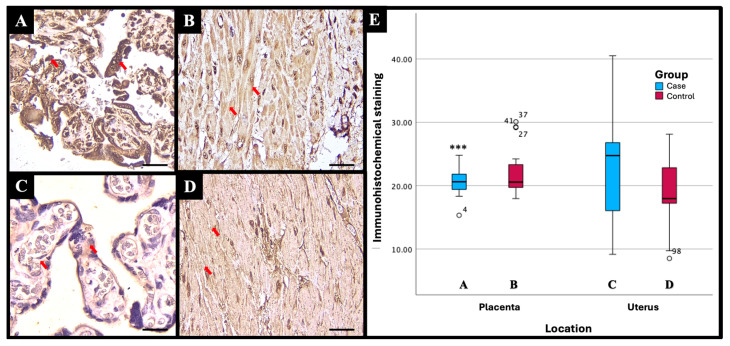
MMP-9 immunohistochemical staining results with different intensities in (**A**) placenta of PASD group: strong, (**B**) uterus of PASD group: moderate, (**C**) placenta of non-PASD group: moderate, and (**D**) uterus of non-PASD group: moderate. MMP-9 expression is characterized by brown staining in the cytoplasm and cell membrane (red arrow); light blue staining indicates hematoxylin-stained nuclei. Note the increased MMP-9 staining in the intervillous spaces compared to control tissues, suggesting enhanced proteolytic activity associated with PASD. (**E**) Comparison of mean immunostaining expression levels (percentage of positive cells) of MMP-9 in PASD and non-PASD groups. ***Mann–Whitney U test, *p* < 0.05 compared with the control group. Scale bar 500 µm; original magnification 400×. This figure was generated using Microsoft PowerPoint, Microsoft, Washington, DC, USA. All images presented were obtained exclusively for this study and have not been previously published or used elsewhere.

Based on the results of the immunohistochemical (IHC) examination, there were significant differences in immunostaining expression levels in several tissues in the PASD group compared with the non-PASD group. The expression of CXCR2, MMP-2, and MMP-9 was characterized by brown staining in the cytoplasm and cell membrane, while purple staining showed hematoxylin and eosin-stained nuclei. The histology picture of the tissue before IHC staining is shown in Figure 1, while the IHC results are shown in Figure 2, Figure 3 and Figure 4.

Immunohistochemical staining with an anti-CXCR2 antibody showed different expression levels between PASD and non-PASD groups in placental and uterine tissues. In the PASD group, both the placenta and uterus had CXCR2 protein expression. However, there was no expression of CXCR2 protein in the placenta or uterus in the non-PASD group (Figure 2).

Different expression levels between PASD and non-PASD groups in placental and uterine tissues were obtained in immunohistochemical stains with the anti-MMP-2 antibody. In the PASD group, both the placenta and uterus were found to have MMP-2 protein expression. However, there was no MMP-2 protein expression in the placenta or uterus in the non-PASD group (Figure 3).

Immunohistochemical staining with the anti-MMP-9 antibody showed different expression levels between PASD and non-PASD groups in placental and uterine tissues. In the PASD group, both the placenta and uterus had MMP-9 protein expression. However, there was no MMP-9 protein expression in the placenta or uterus in the non-PASD group (Figure 4).

There were significant differences in the immunostaining expression levels and IHC score of CXCR2 in placental tissue and CXCR2 and MMP-9 in uterine tissue in the PASD group compared with the non-PASD group (Table 2).

## 4. Discussion

A diagnostic study with a case–control design was conducted to analyze the relationship of the expression of CXCR2, MMP-2, and MMP-9 to the incidence of PASD. There was no significant difference in the general characteristics of the study subjects between the two groups. Matching the general characteristics of subjects in the case and control groups is important to prevent the influence of confounding variables that could potentially affect the results of this study. Research by Liang et al. [13] reported that a BMI with an odds ratio (OR) of 1.04 and 95% confidence interval (CI) of 1.02–1.06, parity (OR = 1.60; 95%CI: 1.42–1.79), and the number of previous cesarean deliveries (OR = 2.57; 95%CI: 2.02–3.26) were independent risk factors for the incidence of PASD in the group of pregnant women with a history of a previous cesarean section. Research by Zhang et al. [14] reported that a maternal age >35 years increased the risk of PASD (OR = 1.26; 95%CI 1.14–1.40). Research by Kilicci et al. [15] also reported that pregnant women with diabetes and hypertension had 3.83 and 29.7 times the risk of PASD, respectively. In this study, there were no significant differences in the variables of maternal age, BMI, parity, number of previous cesarean deliveries, and comorbidities between the case and control groups, so these four variables were not confounding variables that could potentially affect the results of this study.

The gestational age in the case group of 35 (30–39) weeks was significantly lower than the control group’s 37.5 (34–40) weeks (*p* = 0.005). This is in accordance with the results of the research of Shi et al. [16], who reported a lower gestational age in the case group (37.9 ± 1.9 weeks) compared with the control group (36.1 ± 5.3 weeks, *p* < 0.001). This is because the management of PASD with the termination of pregnancy by an elective cesarean section was carried out at a lower gestational age of 34–36 weeks [17].

Based on the results of immunohistochemical (IHC) examination, there were significant differences in immunostaining expression (percentage of positive cells) and the IHC score of CXCR2 in placental tissue and CXCR2 and MMP-9 in uterine tissue in the PASD group compared with the non-PASD group. No previous research has been conducted to evaluate the expression of CXCR2 in the uterine and placental tissues of patients with PASD. Wu et al. [11] examined the level of CXCR2 expression in the placental tissue of 38 patients with pre-eclampsia, comparing it to 21 normal pregnancies. The expression of CXCR2 was reduced in the placental tissue of patients with pre-eclampsia compared to those with a normal pregnancy. This was evident from the lower intensity of immunostaining observed in the cytoplasm of villous syncytiotrophoblast cells and decidual cells. CXCR2 overexpression in PASD is likely to result in higher levels of immunostaining expression compared with the control (normal pregnancy). CXCR2 expression has a role in increasing trophoblast invasion, vascular remodeling, and neovascularization [10]. The study by Ma et al. [12] also reported that CXCR2 as a CXCL1 receptor promotes endothelial cell proliferation, migration, and angiogenesis and plays a role in decidual angiogenesis during the first trimester of pregnancy. The continued study of these biomarkers may bring researchers to light regarding the pathogenesis of PASD.

The currently existing explanation concerning the cause of PASD is that a malfunction in the interface between the endometrium and myometrium results in the inability of the uterine scar area to undergo normal decidualization, hence permitting deeper infiltration of trophoblasts. Therefore, cesarean section wound healing is believed to be a contributing factor in the occurrence of PASD. Cell migration on the extracellular matrix (ECM) and the modification and destruction of the ECM by MMPs are crucial components of the wound healing process. Matrix metalloproteinases are a class of enzymes that include zinc (Zn) and require calcium for their activity. They play a role in breaking down the ECM. MMP-2 has a function in wound healing by speeding up the movement of cells, whereas MMP-9 is produced by keratinocytes at the forefront of the wound to enhance cell movement and the process of re-epithelialization. MMP-2 and MMP-9 are detected in the damaged epithelium [7].

Matrix metalloproteinase is considered as a crucial enzyme involved in the penetration of trophoblast cells during the initial stages of pregnancy. Additionally, it seems to have a role in the invasion of trophoblast cells. Research by El-Hussieny et al. [9] on 26 PASD and 31 non-PASD reported that MMP-2 staining in the cytoplasm of trophoblast villus and extravillous placental tissue of PASD patients was significantly higher than normal placenta. Most PASD cases showed moderate (30.8%) or strong (42.3%) staining, while most control cases showed negative (32.3%) or weak (38.7%) staining. Research by Chen et al. [8] examined MMP-9 expression in 10 PASD patients and 10 non-PASD patients. The positive level of MMP-9 expression in the PASD group (60%) was significantly higher than in the control group (10%). MMP-9 was mainly localized in the cytoplasm of trophoblasts with positive brown granulators or punctata. Placenta accreta is characterized by a reduction in E-cadherin levels and an increase in MMP-9 levels. Trophoblasts are believed to increase trophoblast invasion in accreta [8].

The IHC score was calculated from the staining intensity and the percentage of positive cells stained. In this study, there was a significant difference in the CXCR2 IHC score in placental tissue and CXCR2 and MMP-9 in uterine tissue in the PASD group compared with the non-PASD group. However, due to the small sample size, the results of this study should be interpreted with caution. Further studies with larger sample sizes are needed to assess the expression of CXCR2, MMP-2, and MMP-9 in uterine and placental tissues of PASD patients.

Trophoblast cell implantation and invasion are closely related to the expression of MMPs. Gelatinases, namely MMP-2 and MMP-9, are key in the invasion process. MMP-2 and MMP-9 are expressed differently in trophoblast cells. MMP-2 is the main regulator of trophoblast invasion in early pregnancy. MMP-2 is localized at the base of the placenta during early pregnancy and is dominant over trophoblast MMP-9 at 6–8 weeks gestation. MMP-2 is more abundantly secreted in early pregnancy until week 9. MMP-2 is then continuously expressed throughout pregnancy, but its activity is reduced in the placenta at term gestation. MMP-9 is mainly expressed by trophoblast cells after week 9 [18,19].

Overexpressed laminin subunit gamma 2 (LAMC2) promotes excessive trophoblast invasion via the phosphatidyl inositol-3 kinase (PI3K)/AKT serine/threonine kinase (Akt)/MMP-2/9 pathway in PASD pathogenesis [20]. However, the increase in MMP-9 expression is independent of the increase in MMP-2 [21]. The expression of MMP-2 is constant and most proinflammatory stimuli do not produce a rise in MMP-2 expression because the MMP-2 gene does not include binding sites for proinflammatory transcription factors like activator protein 1. The activation of MMP-2 is crucial for its role in promoting angiogenesis and invasion. However, the activation of MMP-9 occurs through the release of the MMP-9 prodomain by serine proteases or other MMPs. MMP-9 can be activated through alternative pathways in response to oxidative stress, which affects cysteine activation. The stress-activated mitogen-activated protein kinase (MAPK) pathway plays a role in the increased expression of MMP-9 in human trophoblast cells. Tumor necrosis factor (TNF)-α might activate two different pathways leading to MMP-9 expression, namely the extracellular signal regulated kinase (Erk)-1/2 pathway, which then initiates nuclear factor-kappaB (NF-κB) activation, as well as the stress-activated protein kinase (SAPK)/c-Jun N-terminal kinase (JNK) pathway, which activates AP-1 [22]. In addition, an in vivo study by Costanzo et al. [21] reported an increase in MMP-2 levels five hours after injury that reached the highest levels on day 7 and then rapidly decreased. On the other hand, MMP-9 levels remained elevated even after 2 weeks from injury. This is thought to be the reason why MMP-2 in this study did not increase along with the increase in MMP-9 [23].

### 4.1. Clinical Implications

The findings from our study underscore the significant role of CXCR2, MMP-2, and MMP-9 in the pathogenesis of PASD, with potential implications for the early diagnosis and management of this serious pregnancy complication. Our results reveal elevated expressions of CXCR2 and MMP-9 in placental and uterine tissues of patients with PASD, suggesting their involvement in the abnormal placental invasion characteristic of the disorder.

The identification of these biomarkers could transform current diagnostic approaches by providing a molecular basis for early detection. Elevated CXCR2 and MMP-9 levels could potentially be detected through non-invasive methods, such as maternal blood tests, early in pregnancy. This could enable the stratification of risk for PASD in women, particularly those with a history of cesarean delivery or other uterine surgeries, allowing for personalized monitoring and management plans. Early diagnosis could lead to optimized timing of delivery and preparation for potential complications, thereby reducing the morbidity associated with emergency situations during labor and delivery.

Furthermore, understanding the molecular mechanisms by which CXCR2 and MMP-9 contribute to PASD opens new avenues for therapeutic interventions. For instance, targeting CXCR2 signaling pathways or inhibiting MMP-9 activity could be explored as strategies used to limit excessive trophoblastic invasion and prevent the establishment or progression of PASD. These therapeutic approaches could be particularly valuable in cases where early intervention might alter the course of the disease, potentially reducing the need for invasive surgeries such as hysterectomy, which is often required in severe cases.

Future studies should focus on the longitudinal assessment of these biomarkers throughout pregnancy to better understand their dynamics and predictive value. Additionally, experimental models could be employed to test the efficacy of targeted therapies that modulate the activity of CXCR2 and MMP-9, further clarifying their roles in PASD pathology.

In conclusion, our study contributes to a deeper understanding of the molecular underpinnings of PASD and highlights the potential of CXCR2 and MMP-9 as biomarkers for early diagnosis and targets for therapeutic intervention. By advancing our knowledge in these areas, we can improve the clinical outcomes for women afflicted with this challenging condition, ultimately enhancing maternal and fetal health.

### 4.2. Strengths and Limitations of This Study

This is the first study to simultaneously assess clinical, laboratory, and immunohistochemical parameters to predict the incidence of PASD. In addition, the study population included pregnant women with a history of cesarean delivery, which is known to be a major risk factor for PASD. No previous studies have reported the diagnostic value of the PAI score in a population of pregnant women without placenta previa. In addition, few studies have been conducted on PAI scores prospectively. This study is also the first study to describe the expression of CXCR2 in placental tissue as well as the expression of CXCR2, MMP-2, and MMP-9 in uterine tissue of PASD patients. The modified PAI score scoring system consisting of zinc, lacunae, and myometrial thickness parameters in the smallest sagittal section of this study can be further developed to be used in clinical practice to help to confirm the diagnosis of PASD.

This study was a case–control study conducted at only one hospital (single center). In addition, only patients with histopathologically confirmed diagnoses were included in the PASD group, resulting in a small sample size. Furthermore, the findings of this study are relevant in women who have previously undergone cesarean delivery, as all PASD events in our sample were observed in women with that specific risk factor. Pregnant women without a history of cesarean delivery but with other predisposing factors such as a history of uterine surgery were not included in this study. This study also did not examine other biomarkers that may play a role in PASD. Extensive multicenter studies with larger sample sizes are needed to generalize the results and to determine whether combining various other clinical and laboratory parameters can improve accuracy in identifying PASD.

Future studies should include Western blotting and RT-PCR to validate the findings from our immunohistochemical analyses. This multidimensional approach will strengthen our understanding of the roles of these biomarkers in PASD. We also recommend expanding the sample size to include a broader patient population across multiple centers. This approach will enhance the external validity of the findings and may allow for the investigation of additional variables and subgroup analyses that were not feasible in the current study. Furthermore, collaborations with larger healthcare centers or multicenter studies could be particularly beneficial for increasing the sample diversity and the statistical power necessary to explore more subtle associations within the data.

## 5. Conclusions

The expression level of CXCR2 immunostaining in placental and uterine tissues of patients with PASD was significantly different from patients without PASD. The expression level of MMP-2 immunostaining in placental and uterine tissues of patients with PASD was not significantly different from patients without PASD. The MMP-9 immunostaining expression level of PASD patients was significantly different from that of patients without PASD in uterine tissue, but not in placental tissue.

## Figures and Tables

**Table 1:** Characteristics of subjects.

Variables	Mean ± SD Median (Min–Max) *n* (%)	Group		*p* Value	Odds Ratio (95%CI)
		PASD (*n* = 17)	Non–PASD (*n* = 34)		
Maternal age	31.82 ± 4.68	31.82 ± 4.26	31.82 ± 4.93	1.000 ^a^	
20–35 years	37 (72.5)	13 (76.5)	24 (70.6)	0.749 ^d^	0.738 (0.141–3.266)
>35 years	14 (27.5)	4 (23.5)	10 (29.4)		
BMI (kg/m)^2^	26.83 ± 3.27	25.60 ± 2.65	27.44 ± 3.42	0.057 ^a^	
Normal	3 (5.9)	2 (11.8)	1 (2.9)		
Overweight	3 (5.9)	2 (11.8)	1 (2.9)	1.000 ^d^	1 (0.033–29.807)
Obesity I	35 (68.6)	11 (64.7)	24 (70.5)	0.249 ^d^	0.229 (0.019–2.804)
Obesity II	10 (19.6)	2 (11.8)	8 (23.5)	0.154 ^d^	0.125 (0.007–2.176)
Parity	1.80 ± 0.69	1.82 ± 0.52	1.79 ± 0.77	0.888 ^a^	
Primiparity	17 (33.3)	4 (23.5)	13 (38.2)	0.294 ^c^	0.497 (0.098–2.122)
Multiparity	34 (66.7)	13 (76.5)	21 (61.8)		
Gestational age	37 (30–40)	35 (30–39)	37.50 (34–40)	**0.005 ^b^**	
Preterm	24 (47.1)	13 (76.5)	11 (32.4)	**0.003 ^c^**	6.795 (1.554–34.098)
Fullterm	27 (52.9)	4 (23.5)	23 (67.6)		
Comorbidity					
Yes	2 (3.9)	1 (5.9)	1 (2.9)	1.000 ^d^	2.063 (0.025–166,710)
No	49 (96.1)	16 (94.1)	33 (97.1)		

^a^ Independent T-test, ^b^ Mann–Whitney U test, ^c^ Pearson chi-square test, ^d^ Fisher’s exact test. Bold signifies that the *p* value is <0.05

**Table 2:** Comparison of immunostaining expression levels and IHC score in PASD and non-PASD groups.

	Immunostaining Expression Level Mean ± SD				IHC Score Median (Min–Max)			
	Overall	PASD (*n* = 17)	Non-PASD (*n* = 34)	*p* Value	Overall	PASD (*n* = 17)	Non-PASD (*n* = 34)	*p* Value
Placental CXCR2	10.22 ± 3.54	12.24 ± 3.25	9.21 ± 3.28	**0.003 ^a^**	0 (0–4)	2 (1–4)	0 (0–1)	**<0.001 ^b^**
Uterine CXCR2	6.82 ± 2.92	8.70 ± 1.78	5.88 ± 2.94	**<0.001 ^a^**	1 (0–4)	1 (0–4)	0 (0–2)	**<0.001 ^b^**
Placental MMP-2	6.68 (1.25–13.62)	7.15 (3.78–13.62)	6.59 (1.25–12.05)	0.826 ^b^	1 (0–4)	1 (1–4)	1 (0–4)	0.612 ^b^
Uterine MMP-2	14.50 (9.15–22.36)	14.15 (9.76–22.36)	16.00 (9.15–22.34)	0.238 ^b^	2 (1–6)	4 (1–6)	2 (1–6)	0.324 ^b^
Placental MMP-9	20.59 (15.33–30.07)	20.61 (15.33–24.81)	20.56 (17.97–30.07)	0.523 ^b^	4 (2–6)	4 (2–6)	4 (2–6)	0.902 ^b^
Uterine MMP-9	18.31 (8.52–40.50)	24.75 (9.16–40.50)	17.96 (8.52–28.12)	**0.018 ^b^**	4 (1–6)	4 (2–6)	4 (1–4)	**<0.001 ^b^**

^a^ Independent T-test, ^b^ Mann–Whitney U test, α = 0.05. CXCR2: CXC motif chemokine receptor 2; MMP: matrix metalloproteinase; SD: standard deviation; PASD: placenta accreta spectrum disorder. The IHC score was calculated from the staining intensity and the percentage of positive cells stained. Bold signifies that the *p* value is <0.05.

## Data Availability

The data presented in this study are available on request from the corresponding author.
